# Simplified procedure for efficient and unbiased population size estimation

**DOI:** 10.1371/journal.pone.0206091

**Published:** 2018-10-29

**Authors:** Marcos Cruz, Javier González-Villa

**Affiliations:** Department of Mathematics, Statistics and Computer Science, Univ. of Cantabria, Av. Los Castros 48, E-39005 Santander, Spain; Fred Hutchinson Cancer Research Center, UNITED STATES

## Abstract

Population size estimation is relevant to social and ecological sciences. Exhaustive manual counting, the density method and automated computer vision are some of the estimation methods that are currently used. Some of these methods may work in concrete cases but they do not provide a fast, efficient and unbiased estimation in general. Recently, the *CountEm* method, based on systematic sampling with a grid of quadrats, was proposed. It offers an unbiased estimation that can be applied to any population. However, choosing suitable grid parameters is sometimes cumbersome. Here we define a more intuitive grid parametrization, using initial number of quadrats and sampling fraction. A crowd counting dataset with 51 images and their corresponding, manually annotated position point patterns, are used to analyze the variation of the coefficient of error with respect to different parameter choices. Our Monte Carlo resampling results show that the error depends on the sample size and the number of nonempty quadrats, but not on the size of the target population. A procedure to choose suitable parameter values is described, and the expected coefficients of error are given. Counting about 100 particles in 30 nonempty quadrats usually yields coefficients of error below 10%.

## Introduction

Population sizing is a longstanding problem with a wide range of applications such as security, social sciences and ecology. A population is a finite set of *N* separate items or “particles” of interest, e.g. humans, birds, etc. Several approaches have been taken to address the problem. The traditional density method [1, 2] is widely used by media, police and convention organizers for crowd size estimation, but the estimation usually ignores sampling and relies on imprecise visual estimation. Frequently, bird censuses also lean on visual estimation [3–6] or exhaustive manual counting [7, 8] that is slow, tedious and difficult to verify. Automated computer vision can work in some particular cases with regular patterns on homogeneous backgrounds and non-overlapping particles [9–15]. However, automatic algorithms are generally biased and may show a poor performance [16].

**Fig 1:**
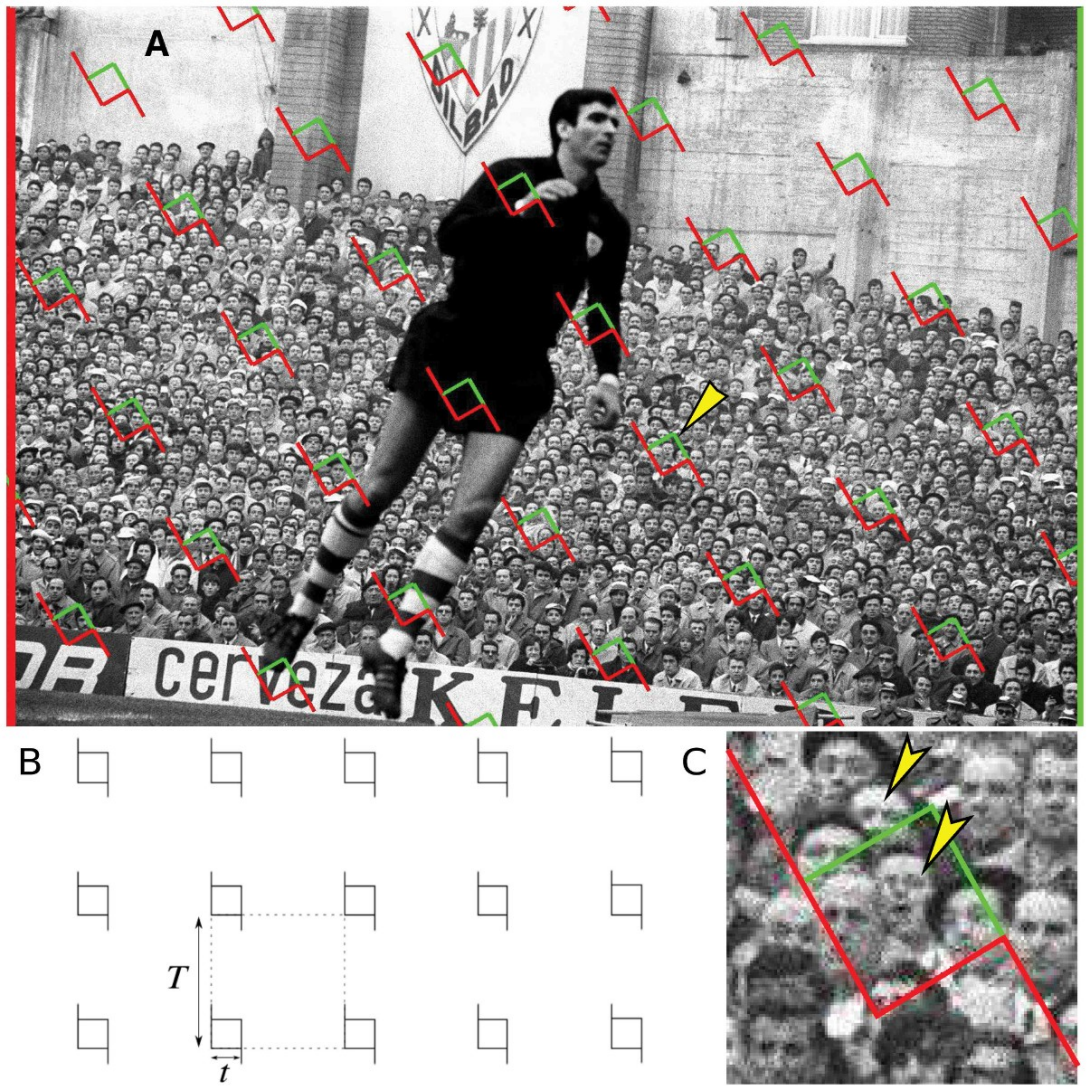
The *CountEm* method. (A): Spectators in a football match (Reprinted from [17] under a CC BY license, with permission from Raúl Cancio, Bilbao, 1966). A square grid of quadrats was superimposed uniformly at random to estimate the total number of spectators in the image. The quadrat marked with a yellow arrowhead is magnified in (C). (B): Square grid of quadrats with parameters *t*, *T*. (C): Forbidden line rule to remove edge effects in manual counting [20]. Only heads marked with yellow arrowheads are counted in the quadrat, the rest are not because they hit the extended forbidden edge (in red).

**Fig 2:**
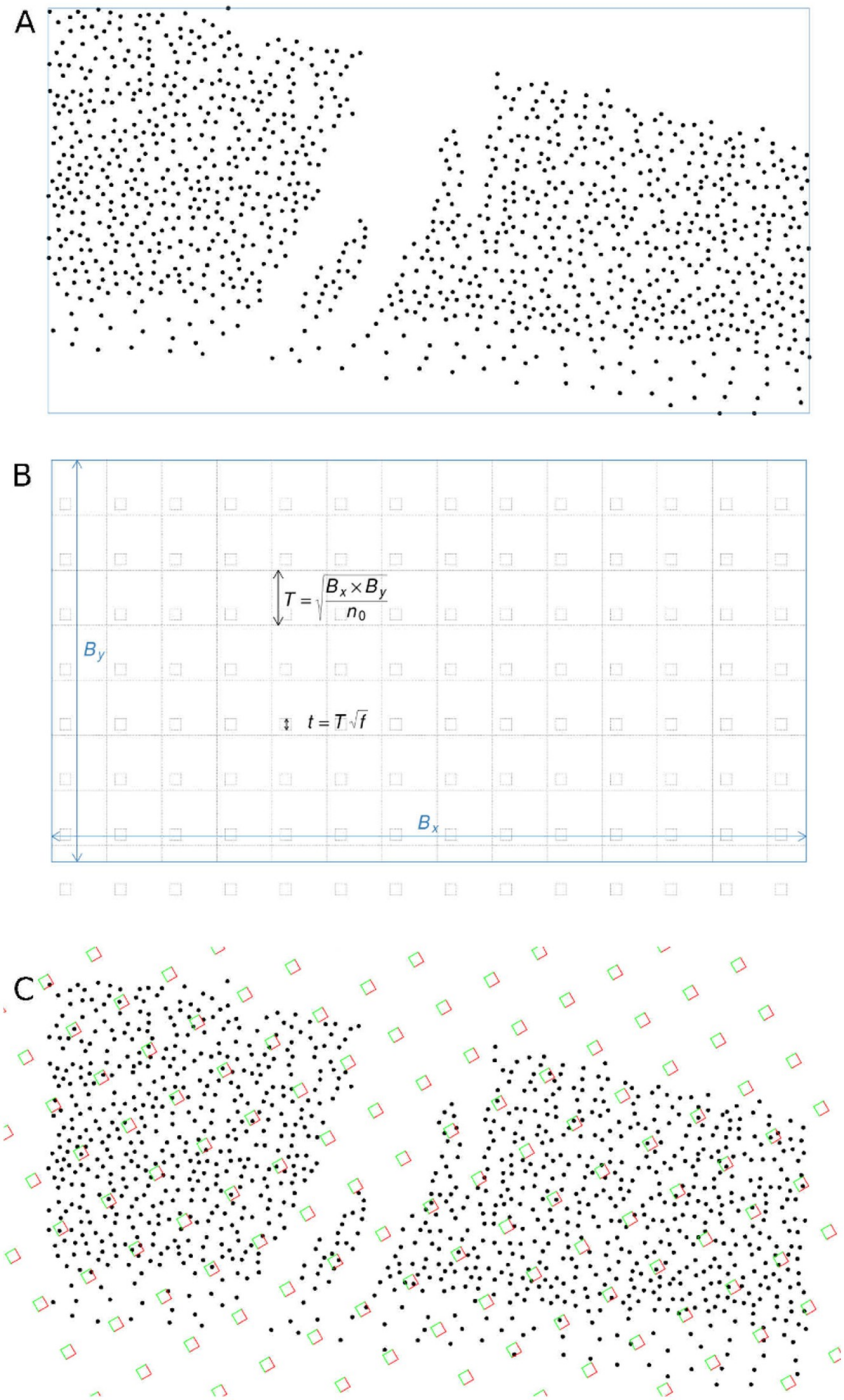
Parameter selection. (A): Point pattern of the *N* = 1120 manually annotated crowd positions corresponding to Fig 1A. The bounding box is plotted in blue. (B): Relation between parameters {*f*, *n*_0_} and {*t*, *T*}. Box size *T* is the area of the bounding box divided by the initial number of quadrats, *n*_0_. Quadrat size, *t*, is the box size times the square root of the sampling fraction, *f*. (C): Random superimposition of the grid of quadrats (B) on point pattern (A). The grid was tilted 30° with respect to the *x* axis.

An unbiased population size estimation method (hereafter *CountEm* method) was recently proposed [17]. *CountEm* can be applied to any kind of particle irrespective of population size and pattern (see for instance Figs 1 and 5 in [17]). The only practical limitation is the basic requirement that all the particles in the population should be unambiguously identifiable for manual counting in the considered image. It is based on well known principles of geometric sampling for stereology which have been previously applied to quantitative microscopy [18, 19]. The main idea is to properly sample and count between 50 and 200 particles in order to estimate populations of any size and spatial distribution. Systematic sampling is performed with a uniform random (UR) grid of quadrats, see Fig 1. The forbidden line rule [20] is used to avoid bias due to edge effects: a particle is counted only if it touches the quadrat but it does not hit the extended forbidden line of the quadrat (see Fig 1C). The population size estimator, $\hat{N}$, is the total number of sampled particles, times the sampling period. The precision of the method was tested [17] on two images with manually annotated particle positions, yielding planar point patterns e.g. Fig 2A. As the method is unbiased, the only source of error comes from sampling variance which can be estimated empirically via Monte Carlo resampling under identical conditions (see Section Simulation procedure). The empirical variance of the population size estimator was computed among 32^2^ = 1024 Monte Carlo replications of the estimator for each given grid of quadrats. The empirical coefficient of error was in the 5% − 10% range, counting about 50 − 100 sampled particles for both point patterns of sizes 1120 and 4633 respectively.

Some practical criteria were given [17] to choose the grid parameters. However, in practice, the choice of starting values for these parameters may not be obvious to every user. Moreover, note that these practical criteria were only checked to be valid on two pictures.

Here we propose to simplify these practical guidelines, by using a more convenient parametrization of the grid. The new estimation protocol is presented and justified in section Materials and Methods. The protocol and the parameter values are tested on the 51 images of the crowd counting dataset, which is described in The crowd counting dataset. The Monte Carlo resampling procedure used to compute the empirical coefficients of error is described in Section Simulation procedure. The results and conclusions are presented in Sections Results and Conclusions respectively.

## Materials and methods

### Definitions and notation

We recall the necessary notation [17]:

- *N*: Population size, i.e. number of particles in the target population.
- $\hat{N}$: Population size estimator.
  $$\begin{array}{c} \hat{N}=\frac{T^{2}}{t^{2}}\cdot Q. \end{array}$$ (1)
- *Q*: Total number of particles sampled by the quadrats.
- *T*: Separation between quadrat centers, *T* > 0.
- *t*: Quadrat side length *t*, (0 < *t* ≤ *T* < ∞).
- *n*: Number of nonempty quadrats.
- ${\text{CE}}_{e}(\hat{N})$: Empirical coefficient of error of $\hat{N}$, calculated via Monte Carlo replications (Eq 7).

Here we propose two alternative grid parameters, *f* and *n*_0_, which are related to *t* and *T* as shown in Fig 2B:

- *f*: Sampling fraction, *f* = *t*^2^/*T*^2^.
- *n*_0_: Initial number of quadrats, *n*_0_ = *B*_*x*_*B*_*y*_/*T*^2^ where *B*_*x*_, *B*_*y*_ represent image width and height in pixels, respectively.

Next we present an outline of the “standard” *CountEm* method [17] and of the simplified protocol which we propose here.

### Outline of the *CountEm* method

The main steps of the standard *CountEm* method [17] are:

1. Crop the image, excluding empty regions as in Fig 2A. This step is optional but highly recommended to increase efficiency.
2. Choose suitable values of *t* and *T* in pixels.
3. Superimpose the grid uniformly at random on the image, e.g. Fig 1A. Optionally, the grid might be tilted at will by a given fixed angle in order to avoid alignments of quadrat and particle rows which would increase the variance [21].
4. Manually count the total number, *Q*, of particles captured by the quadrats. Use the forbidden line rule to ensure unbiasedness: only particles intersecting the quadrat, and not touching the extended forbidden line (in red in Fig 1C), are counted.
5. Use Eq 1 to obtain the estimated population size, $\hat{N}$.
6. Use Eq 3 of our previous paper [17] to predict ${\text{CE}}_{e}(\hat{N})$.

### Outline of the simplified *CountEm* protocol

The simplified *CountEm* protocol proposed here consists of the following steps:

1. Apply standard *CountEm* step 1).
2. Choose suitable values of *f* and *n*_0_. Tentatively one may start with *n*_0_ = 100 and *f* = 0.04.
3. Apply standard *CountEm* step 3).
4. By cursory inspection, check that *Q* and the number of nonempty quadrats are approximately in the following ranges depending on the desired coefficient of error:
  - *Q* ≳ 50 and $n≳20⟹{\text{CE}}_{e}(\hat{N})≲15\%$.
  - *Q* ≳ 100 and $n≳30⟹{\text{CE}}_{e}(\hat{N})≲10\%$.
  - *Q* ≈ 200 and $n\approx 50⟹{\text{CE}}_{e}(\hat{N})\approx 5\%$.
  If *Q* looks too low, then go back to step 2) and increase *f*. On the other hand if *n* is too low, go back to step 2) and increase *n*_0_.
5. Apply standard *CountEm* steps 4), 5) and 6).

The corresponding software is freely available at http://countem.unican.es). The whole estimation process can be made in a few minutes.

### Justification of the protocol

Choosing suitable parameters *t* and *T* in pixels, as suggested in our previous paper [17], can sometimes be laborious. Two practical criteria were given [17], namely aim at (i) having *Q* in the 50 − 150 range, and (ii) counting no more than 4 or 5 particles per quadrat. These two recommendations imply that the number of nonempty quadrats, *n*, may lie between 20 and 50. The resulting coefficient of error should be in the 5% − 10% range.

Consider an example in which the preceding criteria are fulfilled. The goal would be to estimate the number of particles *N* in the image, with coefficient of error below 10% using the old parametrization *t*, *T*. After choosing some initial parameters *t* and *T*, suppose that we count *Q* = 40 in *n* = 18 nonempty quadrats, with an estimated coefficient of error $\text{ce}(\hat{N})=15\%$. In order to reduce the error, we should increase *Q* and *n* since both are below the suggested ranges. But how should we proceed? Increasing *t* to get larger quadrats? Decreasing *T* to obtain more quadrats? Or both? To what extent?

We propose to replace the parameters *t* and *T* with the sampling fraction, *f*, and the initial number of quadrats, *n*_0_ as shown in the preceding subsection. This parametrization is more intuitive and even inexperienced users should find it easy to implement. Reducing the error is straightforward following the simplified procedure with the new parameters as described above.

The validity of the protocol has been checked on 51 images, studying in detail the error ranges corresponding to different sets of parameters.

The empirical squared coefficient of error, ${\text{CE}}_{e}^{2}(\hat{N})$, was computed by Monte Carlo resampling for each of the 51 point patterns in the crowd counting dataset. The dataset is described in Section The crowd counting dataset, whereas the details on the calculation of ${\text{CE}}_{e}^{2}(\hat{N})$ are shown in Section Simulation procedure.

### The crowd counting dataset

**Fig 3:**
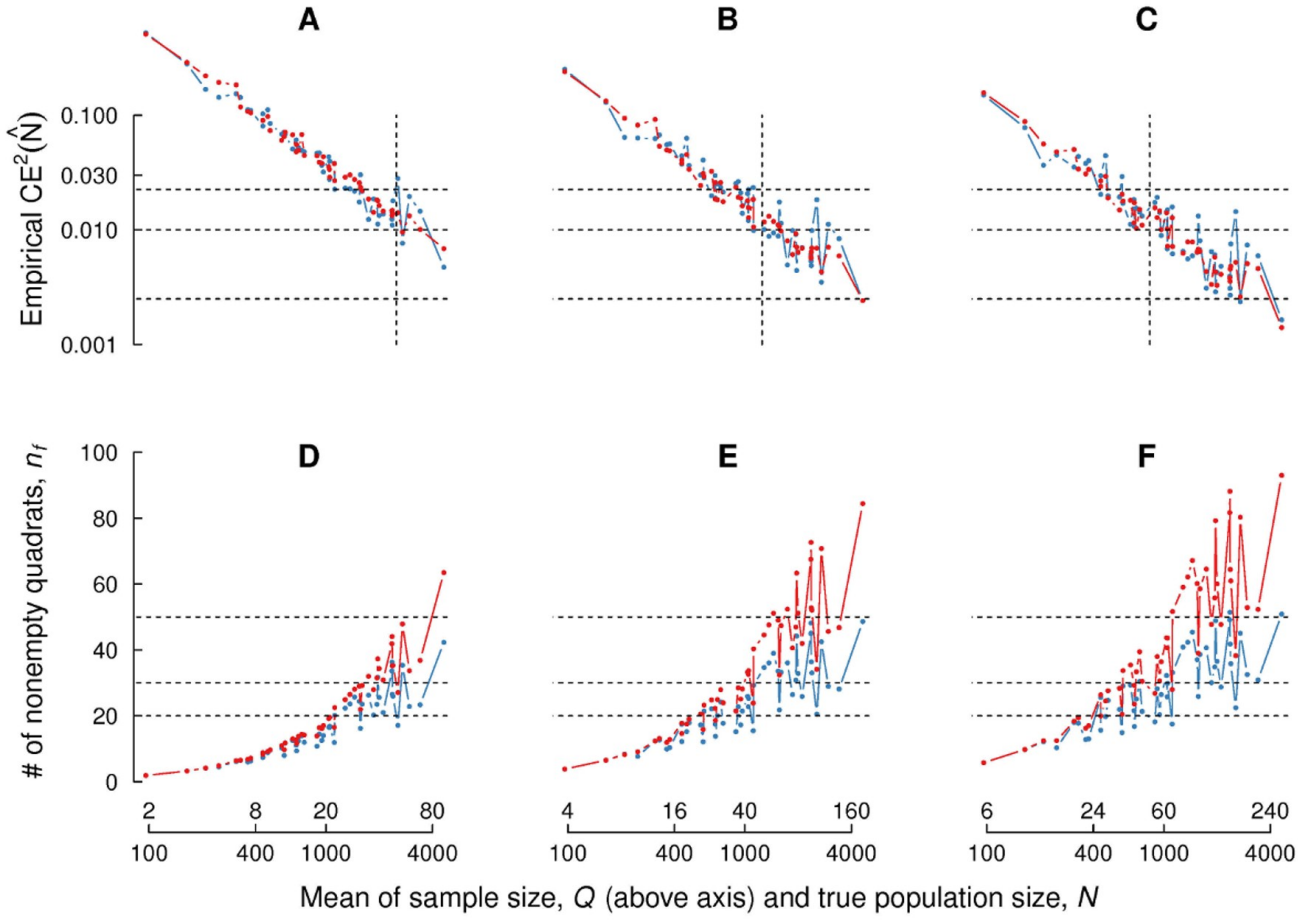
Crowd counting dataset. 15 manually annotated point patterns selected at random from the crowd counting dataset. The total number of point patterns in the dataset is 51.

A total of 51 images were used for two purposes, namely checking the validity of the practical criteria discussed above, and analyzing the optimal *f* and *n*_0_ values to ensure efficient estimation. The crowd sizes, *N*, vary from 96 to 4633. 50 of the images and their corresponding point patterns (see Fig 3) were borrowed from the *UCF dataset* [12]. The additional image is the spectators image countem.unican.es shown in Figs 1 and 2, which was already analyzed in our previous paper [17] together with the image corresponding to the largest crowd of the dataset (*N* = 4633).

### Simulation procedure

The empirical squared coefficient of error ${\text{CE}}_{e}^{2}(\hat{N})$ was computed by Monte Carlo resampling on the 51 point sets for different choices of parameters {*f*, *n*_0_}. The corresponding parameters {*t*, *T*} were calculated as follows (Fig 2B):

$$\begin{array}{ccc} T & = & \sqrt{\frac{B_{x}B_{y}}{n_{0}}},\\ t & = & T\sqrt{f}. \end{array}$$ (2)

The resulting grid was tilted an arbitrary fixed angle of 30° with respect to the *x* axis, before applying the resampling procedure described in our previous paper [17]. Ideally the angle should be suitably selected for each image in order to avoid alignments of quadrat rows with particle patterns. However, visual inspection (Fig 3) reveals that most of the images have either horizontal spectator rows (= 0°) or no concrete particle alignments. Therefore choosing 30° for the whole dataset was judged to be reasonable. This problem was addressed in [21], Fig 11. Next we recall the necessary notation to describe the resampling procedure:

- *Y* = {*y*_1_, *y*_2_, …, *y*_*N*_}: finite set of *N* point particles in a bounded area. We studied 51 such sets (Fig 3).
- *y*_*i*_ ∈ *Y*: *i*th point particle of the set.
- *J*_0_: fundamental square tile or box of side length *T*.
- *z* ∈ *J*_0_: UR point in the fundamental tile.
- Λ_*z*_: UR systematic grid of quadrats, generated by shifting the lower left corner of a quadrat from an arbitrary initial position in *J*_0_ into the UR point *z*, thus dragging the whole quadrat grid together.
- *Q* = *Q*(*Y* ∩ Λ_*z*_): random sample size, namely the total number of particles captured by the quadrats.

For each pair {*t*, *T*} a total of *K*^2^ = 32^2^ = 1024 replicated superimpositions of the grid Λ_*z*_ onto *Y* were generated, corresponding to *K*^2^ systematic replications {*z*_*k*_, *k* = 1, 2, …, *K*^2^} of the point *z* within *J*_0_. These *K*^2^ positions were arranged in a random subgrid within *J*_0_ which should be expected to be more efficient than independent random replications [17]. For each *k*, the corresponding sample total,

$$Q_{k}=Q\left(Y\cap \Lambda_{z_{k}}\right),$$ (3)

was computed automatically from Eq 1 using the *spatstat* package [22]:

$$\begin{array}{c} {\hat{N}}_{k}={\left(T/t\right)}^{2}\cdot Q_{k}. \end{array}$$ (4)

The empirical mean, variance and squared coefficient of error of $\hat{N}$ were computed respectively as follows,

$$\begin{array}{ccc} E_{e}(\hat{N}) & = & K^{-2}\sum_{k=1}^{K^{2}}{\hat{N}}_{k}, \end{array}$$ (5)

$$\begin{array}{ccc} {\text{Var}}_{e}(\hat{N}) & = & K^{-2}\sum_{k=1}^{K^{2}}{\left[{\hat{N}}_{k}-E_{e}(\hat{N})\right]}^{2}, \end{array}$$ (6)

$$\begin{array}{ccc} {\text{CE}}_{e}^{2}(\hat{N}) & = & {\text{Var}}_{e}(\hat{N})/N^{2}. \end{array}$$ (7)

## Results: Justification of the recommended parameter values

**Fig 4:**
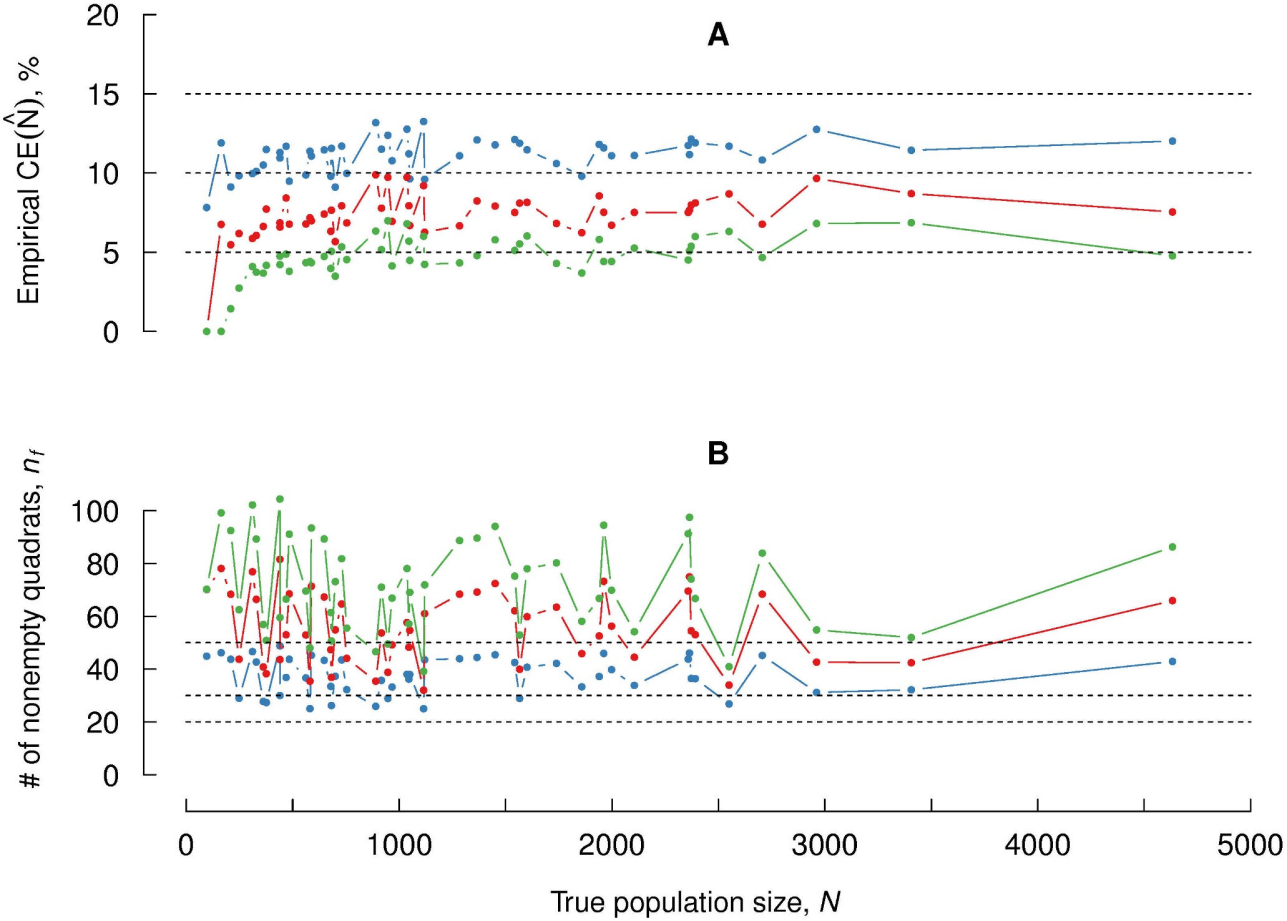
Empirical squared coefficient of error for fixed parameter values. (A, B, C): Empirical squared coefficient of error of the 51 point patterns in the crowd counting dataset, for fixed sampling fractions *f* = 0.02, 0.04, 0.06 respectively. Population and sample sizes are shown on the *x* axis. Blue and red color represent initial number of quadrats *n*_0_ = 50, 100 respectively. Broken horizontal lines correspond to 5%, 10% and 15%, whereas the vertical broken is drawn at sample size *Q* = 50. (D, E, F): Analogous plots for nonempty quadrats *n*. Broken horizontal lines correspond to 20, 30 and 50 quadrats.

**Fig 5:**
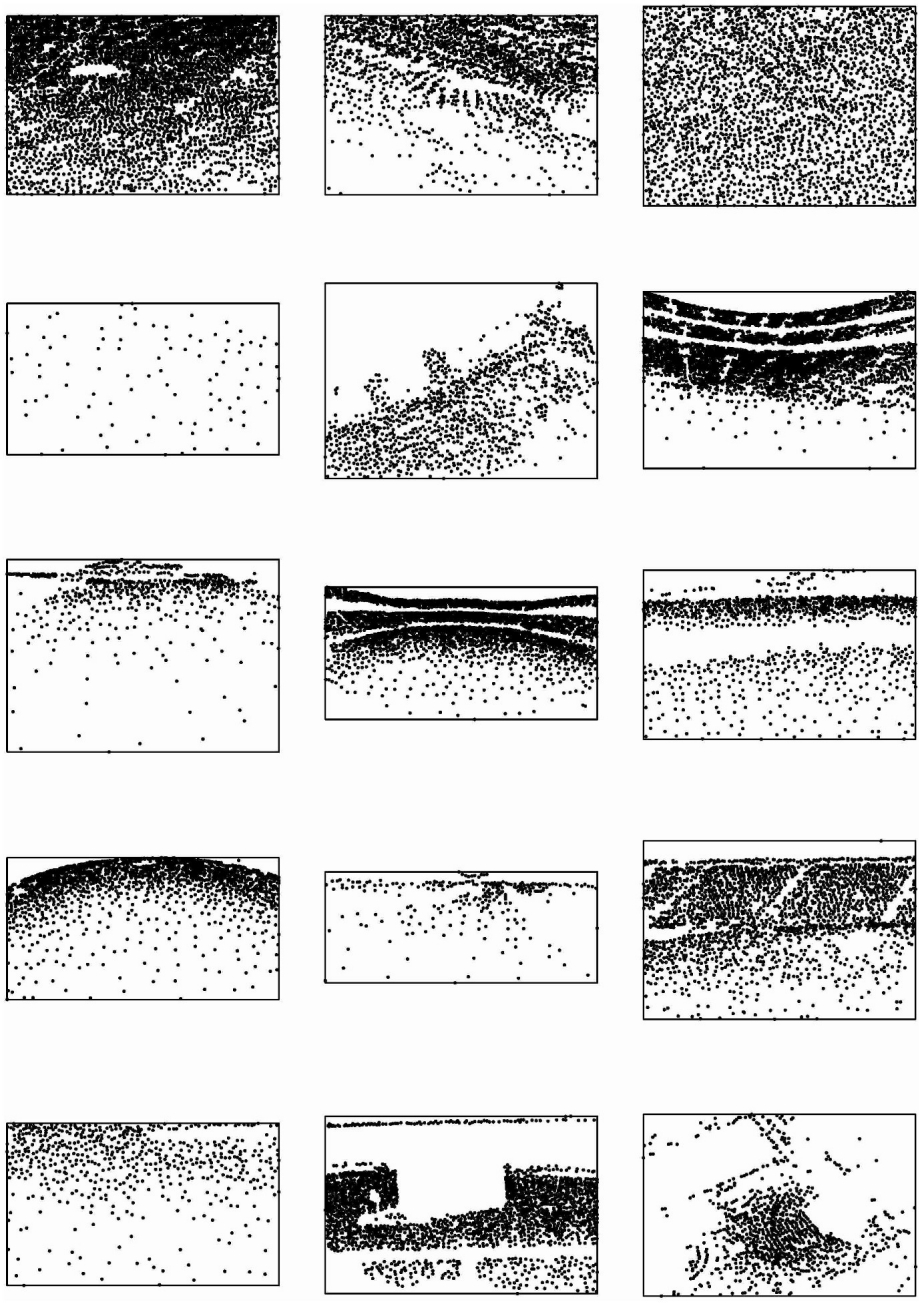
Empirical coefficient of error for optimal parameter values. (A): Empirical coefficient of error, obtained with sampling fractions adapted to each of the 51 point patterns considered in Fig 4. Blue, red and green colors represent sample sizes *Q* = 50, *Q* = 100 and *Q* = 200 respectively. Initial number of quadrats was set to *n*_0_ = 100 for all cases. (B): Analogous plots for nonempty quadrats *n*. The broken horizontal lines are as in Fig 4.

Figs 4 and 5 allow us to justify the tentative values for parameters {*f*, *n*_0_} (step 2) and the practical criteria related to sample size, *Q*, and number of nonempty quadrats, *n*, (step 4) given in the simplified estimation protocol proposed above.

With reference to Fig 4, six different parameter pairs {*f*, *n*_0_} were considered by combining two initial number of quadrats values, namely *n*_0_ = 50, 100 and three sampling fraction values, namely *f* = 0.02, 0.04, 0.06. The empirical squared coefficient of error, ${\text{CE}}_{e}^{2}(\hat{N})$, (top row) and nonempty quadrats, *n*, (bottom row) are represented versus real population size, *N*, and sample size, *Q*. Note that we considered fixed parameter values for all the 51 images, which is not efficient since the resulting *Q* and *n* values depend on population size *N* and spatial distribution of the particles. For instance setting *f* = 0.06 yields *Q* ∼ 6 (too low) for *N* = 96, and *Q* ∼ 278 (too high) for *N* = 4633.

In practice, suitable parameter values should be chosen for each image, but for the present dataset *n*_0_ = 100 works rather well for all images, as shown in Fig 5, where the sampling fraction was selected *a posteriori* as *f* = *Q*/*N*, with *Q* = 50, 100, 200 and *N* the number of manually annotated points in each image. Obviously, this can not be done in practice since *N* is unknown, but the results allow us to establish some tentative values for *f* depending on the order of magnitude of population size *N* and the desired coefficient of error. For instance if we aim at ${\text{CE}}_{e}(\hat{N})≲10\%$ the following starting values can be chosen depending on *N*, aiming at Q≳100:

- $N≲10^{3}⟹f≳0.1$. Note that for population sizes N≲200 the use of *CountEm* is not very helpful since *Q* ∼ *N*. A suitable starting value for small populations is *f* = 0.1.
- $10^{3}≲N≲10^{4}⟹0.1≲f≲0.01$. Therefore *f* = 0.04 could be a reasonable starting value. If the number of particles per quadrat looks higher than 5, a new run with lower *f* is recommended. On the other hand if the resulting *Q* is low, *f* can be increased for a second run.
- $N≳10^{4}⟹f≲0.01$.

There is no upper limit for *N* with *CountEm*, since only *Q* and *n* are relevant for the resulting coefficient of error. Fig 4 shows the approximately linear dependence of ${\text{CE}}_{e}^{2}(\hat{N})$ with respect to *Q*. Counting more than *Q* = 200 particles is not worthwhile in most cases. Fig 5 shows that doubling *Q* from 100 to 200 does only slightly reduce the error.

## Conclusions

*CountEm* describes an unbiased and efficient population size estimation method. It can be used irrespective of population size and pattern. It can be applied to humans, animals, or indeed to any kind of distinguishable particles. We have proposed new parameters to characterize the grid of quadrats, namely sampling fraction *f*, and initial number of quadrats, *n*_0_. A crowd counting data set containing 51 images and corresponding position point patterns have been used to analyze the suitable parameter values and the resulting coefficients of error. Population size has been shown to have no impact on the coefficient of error of the estimation, only sample size, *Q*, and number of nonempty quadrats, *n*, are relevant. Usually Q≳100 and n≳30, yield coefficients of error below 10%. The suitable parameter values depend on the order of magnitude of population size and spatial distribution. For the sizes and spatial distributions of our crowd counting dataset, *n*_0_ = 100 and *f* = 0.04 are reasonable initial values. We believe that the reparametrization defined here allows a more intuitive and fast choice and/or adjustment of the working parameters.

## References

[1] Jacobs H. To count a crowd. Columbia Journalism Review. 1967;6(1):37.

[2] Watson R, Yip P. How many were there when it mattered? Significance. 2011;8(3):104–107. 10.1111/j.1740-9713.2011.00502.x

[3] Sebastián-González E, Green AJ. Reduction of avian diversity in created versus natural and restored wetlands. Ecography. 2016;39(12):1176–1184. 10.1111/ecog.01736

[4] Zhao Q, Silverman E, Fleming K, Boomer GS. Forecasting waterfowl population dynamics under climate change—Does the spatial variation of density dependence and environmental effects matter? Biological conservation. 2016;194:80–88. 10.1016/j.biocon.2015.12.006

[5] Hagy HM, Hine CS, Horath MM, Yetter AP, Smith RV, Stafford JD. Waterbird response indicates floodplain wetland restoration. Hydrobiologia. 2017;804(1):119–137. 10.1007/s10750-016-3004-3

[6] Kingsford RT, Bino G, Porter JL. Continental impacts of water development on waterbirds, contrasting two Australian river basins: Global implications for sustainable water use. Global change biology. 2017;23(11):4958–4969. 10.1111/gcb.13743 28578561

[7] Chabot D, Craik SR, Bird DM. Population census of a large common tern colony with a small unmanned aircraft. PloS one. 2015;10(4):e0122588 10.1371/journal.pone.0122588 25874997 PMC4398491

[8] McEvoy JF, Hall GP, McDonald PG. Evaluation of unmanned aerial vehicle shape, flight path and camera type for waterfowl surveys: disturbance effects and species recognition. PeerJ. 2016;4:e1831 10.7717/peerj.1831 27020132 PMC4806640

[9] Lempitsky V, Zisserman A. Learning To Count Objects in Images In: Advances in Neural Information Processing Systems; 2010 p. 1324–1332.

[10] Descamps S, Béchet A, Descombes X, Arnaud A, Zerubia J. An automatic counter for aerial images of aggregations of large birds. Bird study. 2011;58(3):302–308. 10.1080/00063657.2011.588195

[11] Rodriguez M, Laptev I, Sivic J, Audibert JY. Density-aware person detection and tracking in crowds. In: 2011 International Conference on Computer Vision; 2011. p. 2423–2430.

[12] Idrees H, Saleemi I, Seibert C, Shah M. Multi-Source Multi-Scale Counting in Extremely Dense Crowd Images. In: IEEE Conference on Computer Vision and Pattern Recognition (CVPR). IEEE; 2013. p. 2547–2554.

[13] Botta F, Moat HS, Preis T. Quantifying crowd size with mobile phone and Twitter data. Royal Society Open Science. 2015;2(5):150162 10.1098/rsos.150162 26064667 PMC4453255

[14] Torney CJ, Dobson AP, Borner F, Lloyd-Jones DJ, Moyer D, Maliti HT, et al Assessing Rotation-Invariant Feature Classification for Automated Wildebeest Population Counts. PLOS ONE. 2016;11(5):1–10. 10.1371/journal.pone.0156342 PMC4881999 27227888

[15] Chabot D, Francis CM. Computer-automated bird detection and counts in high-resolution aerial images: a review. Journal of Field Ornithology. 2016;87(4):343–359. 10.1111/jofo.12171

[16] Zhang S, Benenson R, Omran M, Hosang J, Schiele B. How far are we from solving pedestrian detection? In: Proceedings of the IEEE Conference on Computer Vision and Pattern Recognition; 2016. p. 1259–1267.

[17] Cruz M, Gómez D, Cruz-Orive LM. Efficient and Unbiased Estimation of Population Size. PLOS ONE. 2015;10(11):1–14. 10.1371/journal.pone.0141868 PMC4633052 26535587

[18] Howard CV, Reed MG. Unbiased Stereology Three-dimensional Measurement in Microscopy. 2nd ed Oxford: Bios/ Taylor & Francis; 2005.

[19] Cruz-Orive L. STEREOLOGY: A HISTORICAL SURVEY. Image Analysis & Stereology. 2017;36(3):153–177. 10.5566/ias.1767

[20] Gundersen HJG. Notes on the estimation of the numerical density of arbitrary profiles: the edge effect. J Microsc. 1977;111(2):219–223. 10.1111/j.1365-2818.1977.tb00062.x

[21] Gundersen HJG, Jensen EBV, Kiêu K, Nielsen J. The efficiency of systematic sampling in stereology—reconsidered. J Microsc. 1999;193(3):199–211. 10.1046/j.1365-2818.1999.00457.x 10348656

[22] Baddeley A, Rubak E, Turner R. Spatial Point Patterns: Methodology and Applications with R. CRC Press; 2015.

