# Correction: Simplified procedure for efficient and unbiased population size estimation

**DOI:** 10.1371/journal.pone.0208359

**Published:** 2018-11-26

**Authors:** Marcos Cruz, Javier González-Villa

The images for Figs 3, 4 and 5 are incorrectly switched. The image that appears as Fig 3 should be Fig 4, the image that appears as Fig 4 should be Fig 5, and the image that appears as Fig 5 should be Fig 3. The figure captions appear in the correct order.

**Fig 3:**
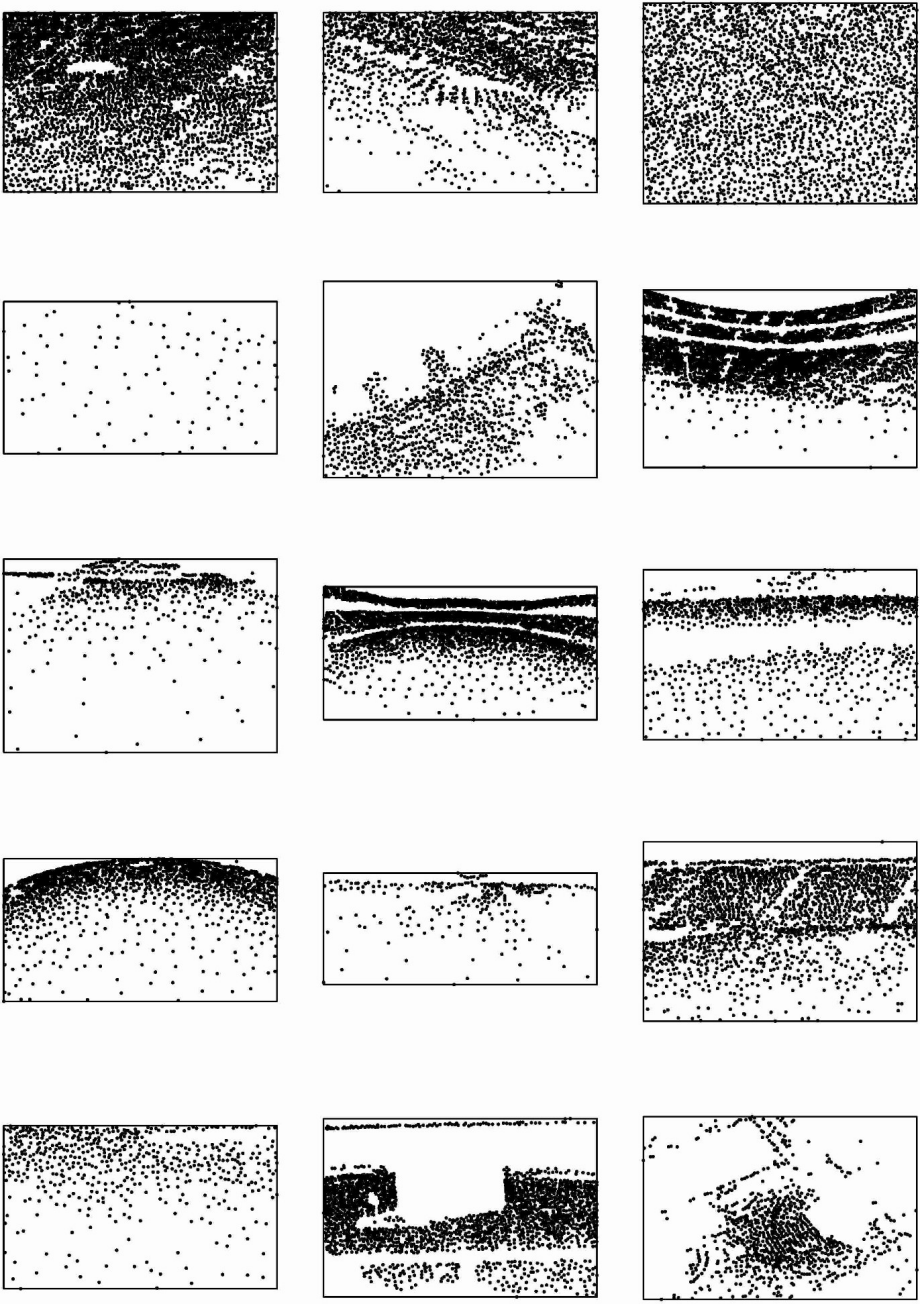
Crowd counting dataset. 15 manually annotated point patterns selected at random from the crowd counting dataset. The total number of point patterns in the dataset is 51.

**Fig 4:**
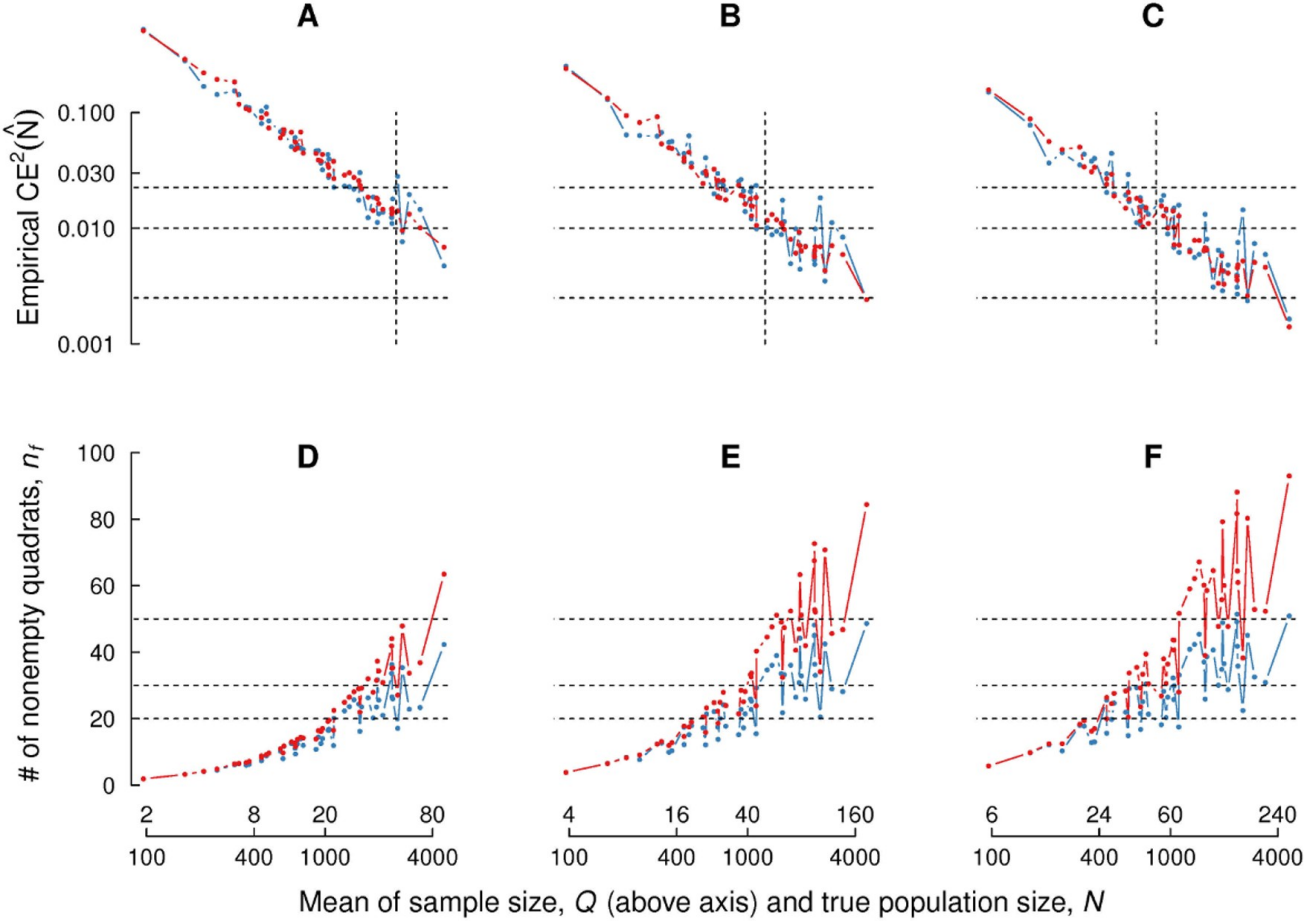
Empirical squared coefficient of error for fixed parameter values. (A, B, C): Empirical squared coefficient of error of the 51 point patterns in the crowd counting dataset, for fixed sampling fractions *f* = 0.02, 0.04, 0.06 respectively. Population and sample sizes are shown on the *x* axis. Blue and red color represent initial number of quadrats *n*_0_ = 50, 100 respectively. Broken horizontal lines correspond to 5%, 10% and 15%, whereas the vertical broken is drawn at sample size Q = 50. (D, E, F): Analogous plots for nonempty quadrats *n*. Broken horizontal lines correspond to 20, 30 and 50 quadrats.

**Fig 5:**
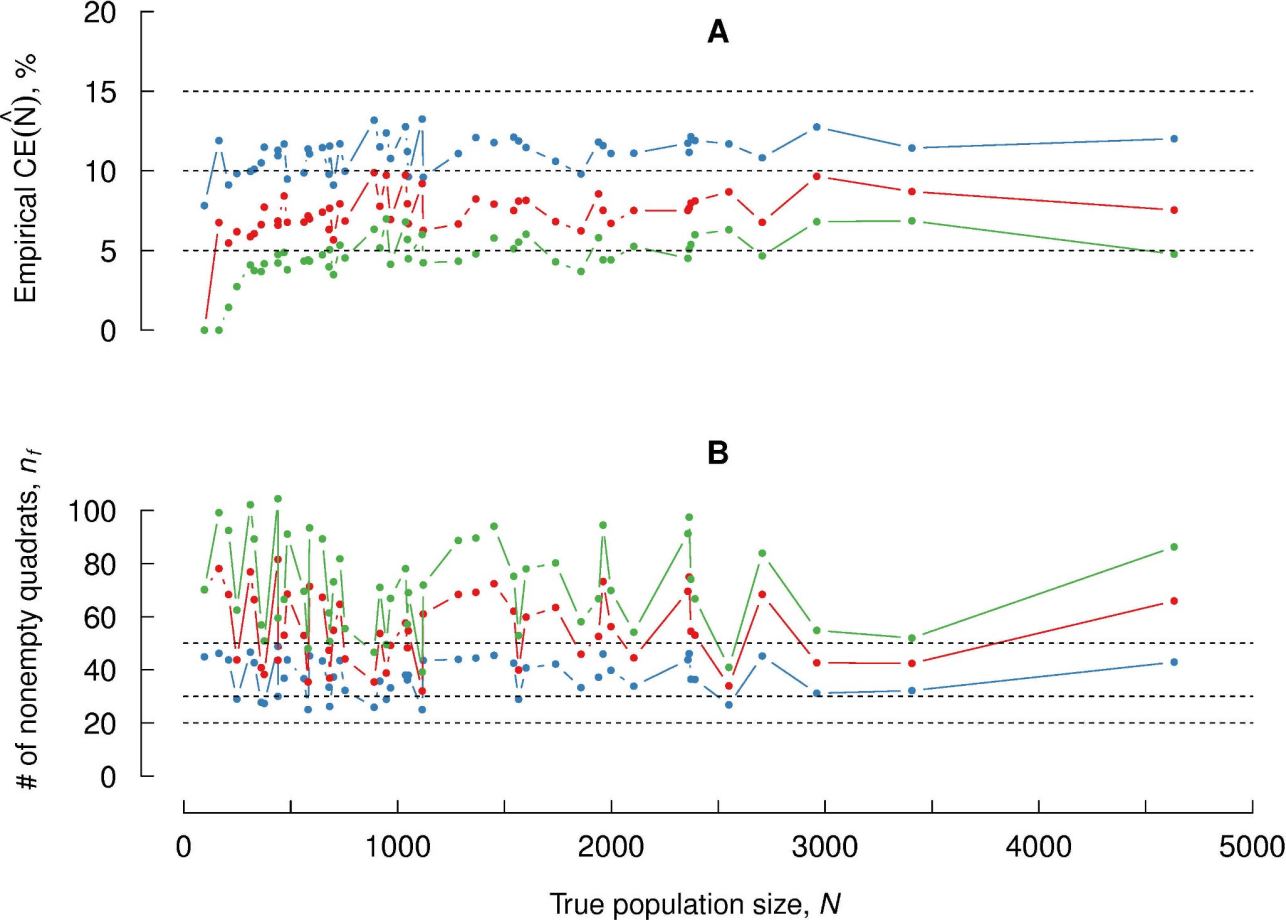
Empirical coefficient of error for optimal parameter values. (A): Empirical coefficient of error, obtained with sampling fractions adapted to each of the 51 point patterns considered in Fig 4. Blue, red and green colors represent sample sizes Q = 50, Q = 100 and Q = 200 respectively. Initial number of quadrats was set to *n*_0_ = 100 for all cases. (B): Analogous plots for nonempty quadrats *n*. The broken horizontal lines are as in Fig 4.
